# Supplementary material for: The causal relationship between the human gut microbiota and pyogenic arthritis: a Mendelian randomization study
Source: Front Cell Infect Microbiol. 2024 Nov 26;14:1452480. doi: 10.3389/fcimb.2024.1452480 (PMC11629706; doi:10.3389/fcimb.2024.1452480)
Supplement: Supplementary file 1 [file DataSheet1.zip › mendelian test/ebi-a-GCST90027591.csv_leave_one_out.pdf]

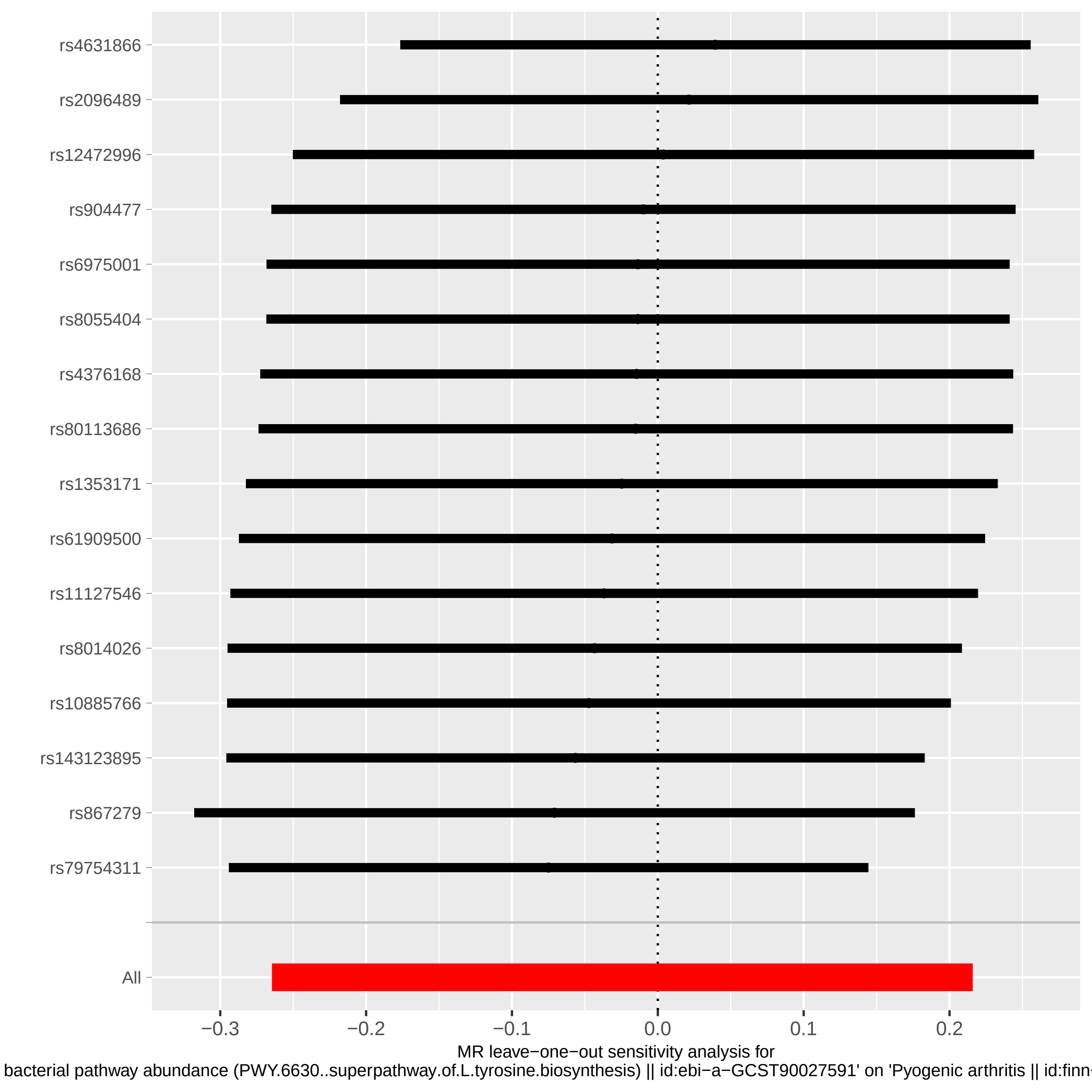

rs4631866
rs2096489
rs12472996
rs904477
rs6975001
rs8055404
rs4376168
rs80113686
rs1353171
rs61909500
rs11127546
rs8014026
rs10885766
rs143123895
rs867279
rs79754311
All
−0.3
−0.2
−0.1
0.0
0.1
0.2
MR leave−one−out sensitivity analysis for
bacterial pathway abundance (PWY.6630..superpathway.of.L.tyrosine.biosynthesis) || id:ebi−a−GCST90027591' on 'Pyogenic arthritis || id:finn-
